# Prognostic Value of *MEG3* and Its Correlation With Immune Infiltrates in Gliomas

**DOI:** 10.3389/fgene.2021.679097

**Published:** 2021-06-16

**Authors:** Xiaoping Xu, Zhenglan Zhong, Yongxiang Shao, Yong Yi

**Affiliations:** ^1^Department of Neurosurgery, The Second People’s Hospital of Yibin, Yibin, China; ^2^Department of Health Examination, The Second People’s Hospital of Yibin, Yibin, China

**Keywords:** *MEG3*, glioma, immune infiltration, prognosis, biomarker

## Abstract

Accumulating evidence has revealed that dysregulated lncRNA expression contributes to the onset and progression of cancer. However, the mechanistic role of lncRNA in glioma progression and tumor immunology remains largely unknown. This study aimed to evaluate the significance of maternally expressed gene 3 (*MEG3*) in the prognosis of and its immune-related roles in gliomas. The expression levels of *MEG3* were analyzed using Oncomine and TIMER database. As an important imprinted gene, the copy number variation (CNV) of *MEG3* in both glioblastoma multiforme (GBM) and low-grade glioma (LGG) were analyzed using GSCALite database, whereas its prognostic significance was assessed using PrognoScan and GEPIA databases. The relationship between *MEG3* and tumor-infiltrated immune cells was analyzed using TIMER. Results showed that *MEG3* expression was lower in most of the human cancer tissues than in the normal tissues. We also found that heterozygous deletion of *MEG3* occurred more frequent than heterozygous amplification in gliomas, and mRNA expression of *MEG3* was significantly positively correlated with its CNV in both the GBM and LGG group. Survival analysis showed that the CNV level of *MEG3* had significant correlation with overall survival (OS) and progression-free survival (PFS) compared with wild type in LGG. Lower *MEG3* expression was related with poor prognosis. Further analysis showed that in GBM, *MEG3* expression level was significantly positively correlated with that of infiltrating CD8^+^ T cells and significantly negatively correlated with that of infiltrating dendritic cells. In LGG, *MEG3* expression level was significantly negatively correlated with levels of infiltrating B cells, CD8^+^ T cells, CD4^+^ T cells, macrophages, neutrophils, and dendritic cells. Univariate Cox survival analysis demonstrated that only the level of infiltrating dendritic cells significantly affected the survival time of patients with GBM, while all six types of immune cells had a significant effect on the survival time of patients with LGG. Furthermore, *MEG3* expression showed strong correlations with multiple immune markers in gliomas, especially in LGG. The current findings suggest that *MEG3* expression might serve as a possible prognostic marker and potential immunotherapeutic target for gliomas.

## Introduction

Glioma is the most common primary malignant tumor of the brain. Despite many breakthrough analyses deciphering the underlying molecular mechanisms of gliomas, comprehensive treatment options are still lacking and the long-term survival rate of glioma patients remains poor. The molecular complexity and unique microenvironment in the brain that lead to therapeutic resistance, disease progression, and tumor recurrence add to the challenges of glioma treatment. Efforts are under way to uncover key molecular mechanisms, which may lead to the development of new therapeutics for glioma patients.

Long non-coding RNA (lncRNA) is an RNA with a length of more than 200 nt. It is known to play an important role in carcinogenesis and cancer progression. Accumulating evidence has revealed that dysregulated lncRNA expression contributes to the development and metastasis of tumors (Pan et al., 2020). However, the mechanistic role of lncRNA in glioma progression and tumor immunology remains largely unknown. Maternally expressed gene 3 (*MEG3*), also known as gene-trap locus 2 (*GTL2*), is an imprinted gene. It is highly expressed in the brain, pituitary gland, placenta, and adrenal gland. *MEG3* has been reported to participate in the regulation of a variety of diseases. The first evidences of the contribution of *MEG3* in human cancers were obtained from pituitary non-functioning adenomas (Zhou et al., 2012). Several studies have shown downregulation of *MEG3* in human cancers, such as breast cancer (Zhang et al., 2016), liver cancer (Zheng et al., 2018), gliomas (Zhang et al., 2017), lung cancer (Wu et al., 2018), squamous cell carcinoma (Sahin et al., 2017), and gastrointestinal cancer (Sun et al., 2014). It was found that *MEG3* expression was significantly reduced or completely lost in 25% of neuroblastomas (Astuti et al., 2005), 81% of hepatocellular cancers (Braconi et al., 2011), and 82% of gliomas (Wang et al., 2012). A recent meta-analysis has also shown a correlation between *MEG3* downregulation and poor patient outcomes (Binabaj et al., 2018).

The tumor microenvironment is now widely accepted as an important regulator of cancer progression and therapeutic response (Klemm et al., 2020). Previous studies have highlighted the association between immune cells and lncRNAs, which are responsible for differentiation, development, and activation of immune cells (Mumtaz et al., 2017). The role of lncRNAs as regulators in the immune modulation of cancer is emerging (Atianand et al., 2017; Denaro et al., 2019). Recent studies indicate that *MEG3* is involved in the regulation of CD4^+^ T cell activation in aplastic anemia (Wang et al., 2019) and the expression level of PD-L1 in aggressive endometrial cancer (Xu et al., 2020). However, to the best of our knowledge, the immune-specific functions of *MEG3* expression in gliomas have not yet been evaluated. The success of immunotherapies (Lim et al., 2018; Schalper et al., 2019) calls for the evaluation of the role of *MEG3* in gliomas. Hence, we conducted the present bioinformatics study to detect the expression levels of *MEG3* in gliomas using Oncomine and “Tumor Immune Estimation Resource (TIMER)” databases, analyze CNV using GSCALite database, and analyze its prognostic significance using PrognoScan and “Gene Expression Profiling Interactive Analysis (GEPIA)” databases. We further investigated the correlation between *MEG3* expression and immune cell infiltration in gliomas by using the TIMER database.

## Materials and Methods

### Gene Expression Analysis

The gene expression level of *MEG3* in various cancers was analyzed using Oncomine (Rhodes et al., 2004)^1^ and TIMER (Li et al., 2017)^2^ databases. The parameters were set as follows in the Oncomine database: *P*-value of 0.001, fold change of 2, and gene ranking of top 10%, six studies meeting the criteria were included for further analysis, including TCGA Brain, Sun Brain (Sun et al., 2006), Murat Brain (Murat et al., 2008), Bredel Brain2 (Bredel et al., 2005), Liang Brain (Liang et al., 2005), and Shai Brain (Shai et al., 2003). These studies reported a total of 955 cases, of which 932 are primary tumor samples, and 904 samples have *MEG3* profile data. We further evaluated the expression level of *MEG3* using TIMER (*P* < 0.05).

### Copy Number Variation (CNV) Analysis

Monoallelic expression dependent on the parent of origin is a hallmark of imprinted genes; it is often lost in certain tumors (Lozano-Urena et al., 2021). We further analyzed the CNV of *MEG3* in both GBM and LGG using GSCALite database (Liu et al., 2018)^3^, which consists of analytic modules for data from three major sources including multi-omics data from TCGA, Genomics of Drug Sensitivity in Cancer (GDSC) (Yang et al., 2013), and Cancer Therapeutics Response Portal (CTRP) (Basu et al., 2013), and normal tissue expression data from GTEx.

### Prognostic Value Analysis

We further analyzed the correlation between *MEG3* expression and clinical prognosis in various types of cancers using the PrognoScan database^4^, which is a powerful tool to investigate the prognostic value of genes (Mizuno et al., 2009). All survival values, such as overall survival (OS), relapse-free survival (RFS), disease-specific survival (DSS), disease-free survival (DFS), and distant metastasis-free survival (DMFS), were included in the present analysis. Only those studies with corrected *P* < 0.05 were included in further analyses. The GEPIA database (Tang et al., 2017)^5^ was used to generate survival curves, including OS, and RFS, based on gene expression using the log-rank test and Mantel–Cox test in both LGG and GBM.

### Immune Infiltration Analysis

Tumor immune estimation resource (TIMER) is a comprehensive database designed for the analysis of immune cell infiltrates across different types of cancers (see text footnote 2) (Li et al., 2017). We initially analyzed the correlation of *MEG3* expression with the abundance of six immune infiltrates, including B cells, CD4^+^ T cells, CD8^+^ T cells, neutrophils, macrophages, and dendritic cells, using the TIMER “gene” module. Furthermore, we used the Kaplan–Meier method to analyze the influence of *MEG3* expression and immune cell infiltration on the prognosis of GBM and LGG patients; we also constructed a multivariate Cox proportional risk model. Lastly, the correlation of *MEG3* expression with different immune infiltrating cell markers was also assessed through the correlation module. These immune infiltrating cells, including T cells, B cells, monocytes, tumor-associated macrophages (TAMs), macrophages, neutrophils, natural killer (NK) cells, dendritic cells, different T-helper cells, Tregs, and exhausted T cells, and the immune cell markers were referenced as described in the previous studies (Danaher et al., 2017; Zhang et al., 2019). The GEPIA database was used to confirm the gene correlations identified by TIMER analysis. The tumor tissue datasets were used for the analysis, and all default setting values were accepted.

### Statistical Analysis

The results generated using the Oncomine and TIMER databases are shown with the *P*-value. Survival curves were generated using PrognoScan and Kaplan–Meier plots. The results of Kaplan–Meier plots and PrognoScan are accompanied with the hazard ratio (HR) and *P* or Cox *P*-values from a log-rank test. The correlation of gene expression was evaluated by Spearman’s correlation and a *P* < 0.05 was considered statistically significant.

## Results

### Analysis of *MEG3* Expression Levels

**FIGURE 1:**
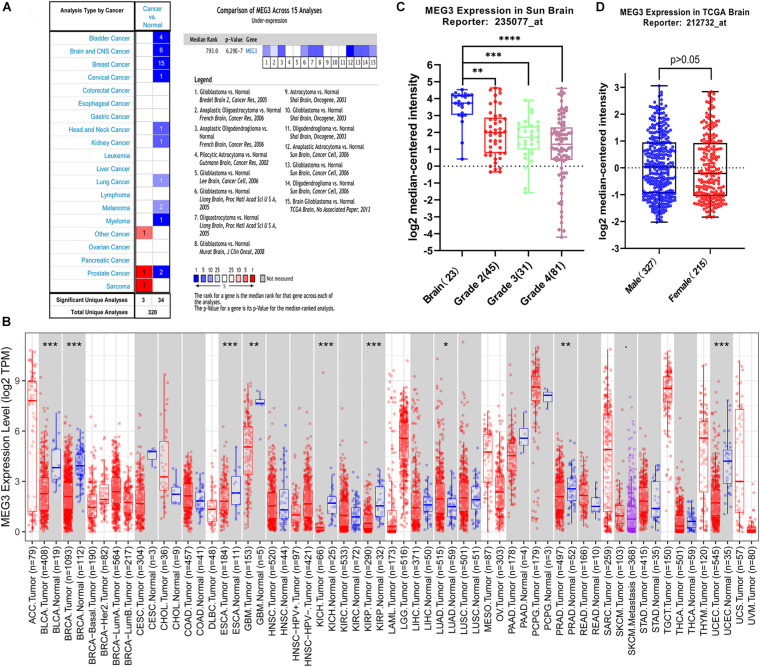
**(A)** MEG3 expression levels in different types of human cancers compared with normal tissues in the Oncomine database. **(B)** Human MEG3 expression levels in different tumor types from TCGA database were determined by TIMER (*P < 0.05, **P < 0.01, ***P < 0.001, ****P < 0.0001). **(C)** The expression of MEG3 decreased with increase of grade. **(D)** Comparison of the expression level of MEG3 in male and female.

*MEG3* expression level in different human cancers and normal tissues were analyzed using Oncomine database. It was observed that *MEG3* expression was lower in most tumor tissues than in normal tissues. In brain and CNS cancers, six studies reported the downregulation of *MEG3*, whereas no study reported its upregulation. A comprehensive analysis of 15 studies yielded a median rank = 793 and *P* = 6.29E-7, indicating that *MEG3* was downregulated in glioma tissues (Figure 1A). Furthermore, we evaluated the *MEG3* expression level through the TIMER database, finding that *MEG3* expression was significantly lower in BLCA (bladder urothelial carcinoma), BRCA (breast invasive carcinoma), ESCA (esophageal carcinoma), GBM (glioblastoma multiforme), KICH (kidney chromophobe), KIRP (kidney renal papillary cell carcinoma), PRAD (prostate adenocarcinoma), UCEC (uterine corpus endometrial carcinoma), than in normal controls. By contrast, *MEG3* expression was significantly higher in LUAD (lung adenocarcinoma) than in normal tissue (Figure 1B). We also further analyzed the expression of *MEG3* in different grades of gliomas, finding that the expression level of *MEG3* in grade 4 was significantly lower than that in other grades of gliomas and normal control, it decreased with the increasing of malignant degree of gliomas (Figure 1C). However, no significant difference was observed with respect to sex (Figure 1D). Finally, the expression of *MEG3* showed a significant difference in LGG and GBM compared with that in normal control through the GEPIA database (Supplementary Figure 1). Taken together, these results were consistent with previous studies that *MEG3* might serve as a tumor suppressor in most human cancers, including gliomas (Ghafouri-Fard and Taheri, 2019).

### Analysis of CNV of *MEG3* Expression

**FIGURE 2:**
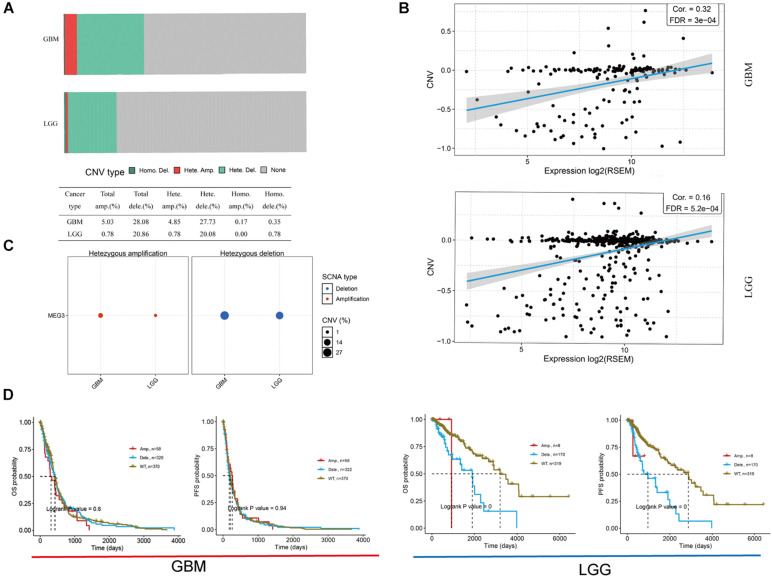
Copy number variation (CNV) of MEG3 expression in both GBM and LGG by using GSCALite database. **(A)** CNV distribution of MEG3 in GBM and LGG tumor samples. **(B)** Spearman correlation between MEG3 CNV and mRNA expression in GBM and LGG. **(C)** Heterozygous CNV in each cancer. **(D)** OS and PFS of MEG3 CNV in GBM and LGG.

Imprinted gene plays an important role in different biological processes, and loss of imprinting has been found in various cancers (Lozano-Urena et al., 2021). By analyzing GSCALite database, we found that the heterozygous deletion of *MEG3* occurred in 27.73 and 20.08% of GBM and LGG, respectively (Figures 2A,C), and Pearson correlation analysis showed that mRNA expression of *MEG3* was significantly positively correlated with its CNV in both GBM (*r* = 0.32, *P* < 0.05) and LGG group (*r* = 0.16, *P* < 0.05) (Figure 2B), which indicated that mRNA expression of *MEG3* was significantly affected by CNV. Survival analysis showed that CNV level of *MEG3* had significant correlation with OS and progression-free survival (PFS) compared with wild type in LGG, but not in GBM (Figure 2D).

### Analysis of the Clinical Prognostic Impact of *MEG3* Expression

**FIGURE 3:**
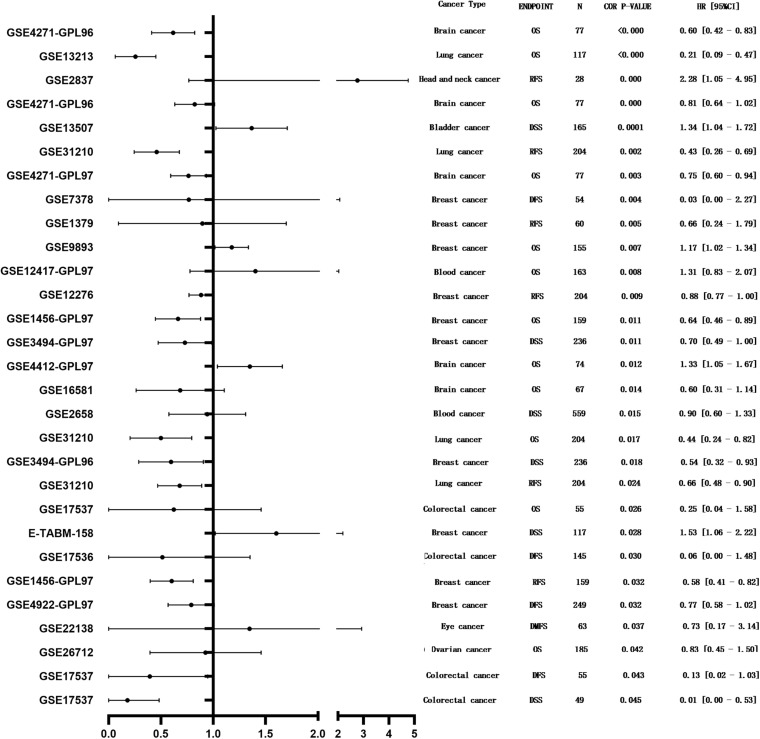
Forest plot displaying the prognostic results for MEG3 generated by using the PrognoScan database.

PrognoScan was used to investigate the potential prognostic impact of *MEG3* expression level in patients suffering from different types of cancer. The results are summarized in Figure 3. Notably, *MEG3* expression was significantly correlated with the prognosis of a total of nine types of cancers, including bladder cancer, brain cancer, breast cancer, head and neck cancer, blood cancer, colorectal cancer, eye cancer, lung cancer, and ovarian cancer. We found that lower *MEG3* expression was often associated with a poorer prognosis in these cancer patients.

We further examined the potential impact of the *MEG3* expression on survival rate in LGG and GBM patients using the GEPIA database. Interestingly, the results illustrated that *MEG3* expression level had no significant correlation with OS and RFS in LGG and GBM (Supplementary Figure 2). These results confirmed the differential prognostic value of *MEG3* in specific types of cancer.

### Analysis of Immune Infiltration

**FIGURE 4:**
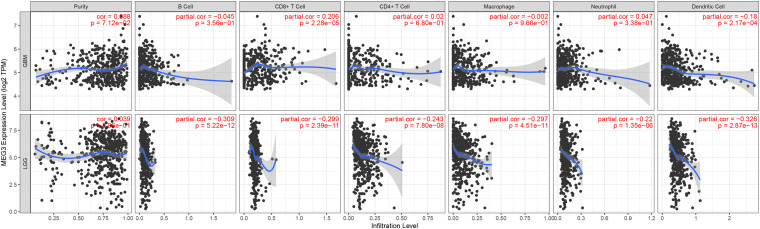
Correlation of MEG3 expression with immune infiltration level in GBM and LGG.

**FIGURE 5:**
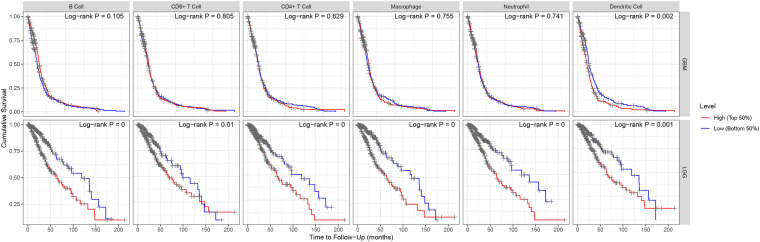
Kaplan–Meier plots of immune infiltration level in GBM and LGG.

“Tumor-infiltrating lymphocytes” are a key predictor of survival as well as response to immunotherapy in the cancer patients (Ohtani, 2007; Azimi et al., 2012). TIMER database was used to evaluate whether the expression of *MEG3* in glioma is correlated with immune infiltration. Interestingly, we found that in GBM, the *MEG3* expression level was significantly positively correlated with level of infiltrating CD8^+^ T cells (*r* = 0.206, *P* = 2.28e-05) and significantly negatively correlated with the level of infiltrating dendritic cells (*r* = –0.18, *P* = 2.17e-04). On the other hand, in LGG, the *MEG3* expression level was significantly negatively correlated with levels of infiltrating B cells (*r* = –0.309, *P* = 5.22-12), CD8^+^ T cells (*r* = –0.299, *P* = 2.39e-11), CD4^+^ T cells (*r* = –0.243, *P* = 7.80e-08), macrophages (*r* = –0.297, *P* = 4.51e-11), neutrophils (*r* = –0.22, *P* = 1.35e-06), and dendritic cells (*r* = –0.326, *P* = 2.87e-13) (Figure 4 and Table 1). Next, to evaluate the correlation between immune cell infiltration and the prognosis of glioma patients, “Survival” module was used to generate Kaplan–Meier plots. Of note, we found that only dendritic cell infiltration was significantly correlated with GBM prognosis, while infiltration of all six types of immune cells showed significant correlation with LGG prognosis (Figure 5). Lastly, to explore the clinical relevance of immune cell subsets in gliomas, Cox proportional hazard model was constructed. It was observed that only dendritic cell infiltration was significantly associated with OS of patients with GBM as revealed by the univariate Cox survival analysis. Six types of immune cells significantly affected the survival time of patients with LGG; however, *MEG3* expression did not significantly affect the survival time in these patients (Table 2). In addition, multivariate Cox survival analysis revealed age, macrophages, *MEG3* expression, and neutrophils to be independent prognostic biomarkers for LGG (Table 3), and age and dendritic cells to be the independent prognostic biomarkers for GBM (Table 4). These findings suggest that *MEG3* plays an important immune-related role in gliomas, particularly in LGG.

**TABLE 1:** MEG3 expression associated with immune infiltration level in GBM and LGG.

Cancer	Variable	Partial.cor	*P*
LGG	Dendritic Cell	–0.32627	2.87E-13
LGG	B Cell	–0.30865	5.22E-12
LGG	CD8^+^ T Cell	–0.29929	2.39E-11
LGG	Macrophage	–0.29677	4.51E-11
LGG	CD4^+^ T Cell	–0.24314	7.80E-08
LGG	Neutrophil	–0.21957	1.35E-06
GBM	CD8^+^ T Cell	0.205579	2.28E-05
GBM	Dendritic Cell	–0.17995	0.000217
GBM	Purity	0.08822	0.071239
GBM	Neutrophil	0.046996	0.337813
GBM	B Cell	–0.0453	0.355574
LGG	Purity	0.038866	0.396039
GBM	CD4^+^ T Cell	0.020212	0.680316
GBM	Macrophage	–0.00212	0.96552

**TABLE 2:** Univariate analysis of the correlation of MEG3 expression and immune infiltrates with OS in glioma.

Cancer	Variable	*P*
LGG	Neutrophil	5.83E-06
LGG	Macrophage	9.20E-06
LGG	B Cell	4.25E-05
LGG	CD4^+^ T Cell	0.00046
LGG	Dendritic Cell	0.000634
GBM	Dendritic Cell	0.001617
LGG	CD8^+^ T Cell	0.009535
GBM	B Cell	0.105243
GBM	MEG3	0.262632
LGG	MEG3	0.541297
GBM	CD4^+^ T Cell	0.629122
GBM	Neutrophil	0.741002
GBM	Macrophage	0.755369
GBM	CD8^+^ T Cell	0.805153

**TABLE 3:** Multivariate analysis of the correlation of MEG3 expression and immune infiltrates with OS in LGG.

	Coef	HR (95%CI_l–95%CI_u)	*P*-value	Sig
Age	0.06	1.062 (1.045–1.079)	0	***
Macrophage	6.607	740.156 (12.152–45,083.396)	0.002	**
MEG3	0.169	1.184 (1.022–1.373)	0.024	*
Neutrophil	-8.367	0 (0–0.956)	0.049	*
CD8_Tcell	7.057	1160.386 (0.832–1,617,980.188)	0.056	
Dendritic	2.432	11.383 (0.177–731.417)	0.252	
Gendermale	0.168	1.183 (0.787–1.776)	0.419	
B_cell	1.697	5.455 (0.008–3945.616)	0.614	
Purity	0.168	1.183 (0.44–3.178)	0.739	
CD4_Tcell	-1.008	0.365 (0–1071.192)	0.805	
raceBlack	15.941	8,377,586.947 (0–Inf)	0.994	
raceWhite	16.156	10,383,164.77 (0–Inf)	0.994	

*P < 0.01; **P < 0.001; ***P < 0.0001.

**TABLE 4:** Multivariate analysis of the correlation of MEG3 expression and immune infiltrates with OS in GBM.

	Coef	HR (95%CI_l–95%CI_u)	*P*-value	Sig
Age	0.029	1.030 (1.021–1.038)	0	***
Dendritic	0.561	1.753 (1.275–2.410)	0.001	**
CD8_Tcell	0.354	1.424 (0.907–2.235)	0.124	
B_cell	-0.48	0.619 (0.323–1.186)	0.148	
RaceWhite	0.551	1.736 (0.705–4.272)	0.23	
Macrophage	0.438	1.550 (0.721–3.331)	0.262	
MEG3	0.099	1.104 (0.891–1.368)	0.366	
Gendermale	0.082	1.085 (0.863–1.365)	0.484	
RaceBlack	0.289	1.335 (0.482–3.701)	0.578	
CD4_Tcell	0.17	1.186 (0.540–2.603)	0.671	
Neutrophil	0.116	1.123 (0.446–2.825)	0.806	
Purity	0.05	1.051 (0.539–2.050)	0.884	

**P < 0.001; ***P < 0.0001.

### Assessment of Correlation Between *MEG3* and Immune Marker Expression

To further investigate the relationship between *MEG3* and level of immune cell infiltration, we assessed the correlation between *MEG3* expression and immune marker genes of various immune cells in glioma using TIMER and GEPIA databases. Different subsets of immune cells, including CD8^+^ T cells, T cells, B cells, monocytes, TAMs, M1 and M2 macrophages, neutrophils, NK cells, and dendritic cells, were analyzed in LGG and GBM, using GBM as the control group. Functional T cells, such as Th1 cells, Th2 cells, Tfh cells, Th17 cells, and Tregs, and exhausted T cells were also analyzed. The results showed that, after adjustment by purity, *MEG3* expression level was significantly negatively correlated with most immune marker sets of various immune cells in LGG. However, interestingly, we found that only dendritic cell markers (HLA-DRA, CD11c) were significantly negatively correlated with *MEG3* expression level in the GBM control group (Table 5). Similar results were also obtained while analyzing the correlation between *MEG3* expression and the above-stated markers by using the GEPIA database (Table 6). Therefore, these results further confirm the findings that *MEG3* is specifically correlated with immune infiltrating cells in gliomas, especially in LGG; this suggests that *MEG3* plays a vital role in immune escape in the glioma microenvironment.

**TABLE 5:** Correlation analysis between MEG3 and relate genes and markers of immune cells in TIMER.

Description	Gene markers	LGG				GBM			
		None		Purity		None		Purity	
		Cor	*P*	Cor	*P*	Cor	*P*	Cor	*P*
CD8^+^ T cell	CD8A	0.085	0.054	0.106	0.021	0.021	0.793	0.019	0.822
	CD8B	–0.180	***	–0.156	***	–0.083	0.308	–0.094	0.277
T cell (general)	CD3D	–0.204	***	–0.178	**	–0.213	*	–0.265	0.018
	CD3E	–0.223	***	–0.213	***	–0.15	0.064	–0.182	0.034
	CD2	–0.25	***	–0.238	***	–0.186	0.021	–0.229	*
B cell	CD19	–0.15	***	–0.136	**	–0.047	0.562	–0.075	0.385
	CD79A	–0.067	0.127	–0.058	0.205	–0.017	0.831	–0.062	0.474
Monocyte	CD86	–0.328	***	–0.359	***	–0.182	0.024	–0.212	0.013
	CD115 (CSF1R)	–0.25	***	–0.27	***	–0.113	0.165	–0.152	0.076
TAM	CCL2	–0.221	***	–0.23	***	–0.11	0.174	–0.112	0.193
	CD68	–0.366	***	–0.379	***	–0.107	0.187	–0.101	0.240
	IL10	–0.257	***	–0.268	***	–0.155	0.056	–0.153	0.075
M1 macrophage	INOS (NOS2)	0.155	**	0.183	***	0.011	0.897	0.016	0.851
	IRF5	–0.2	***	–0.211	***	0.06	0.463	0.065	0.449
	COX2(PTGS2)	0.085	0.055	0.069	0.132	0.015	0.853	0.034	0.689
M2 macrophage	CD163	–0.254	***	–0.258	***	–0.136	0.094	–0.15	0.081
	VSIG4	–0.275	***	–0.292	***	–0.15	0.065	–0.142	0.099
	MS4A4A	–0.341	***	–0.349	***	–0.227	*	–0.226	0.008
Neutrophils	CD66b (CEACAM8)	0.024	0.58	0.034	0.453	0.143	0.078	0.162	0.058
	CD11b (ITGAM)	–0.228	***	–0.25	***	0.018	0.830	–0.001	0.990
	CCR7	–0.092	0.036	–0.078	0.090	–0.084	0.3	–0.113	0.190
Natural killer cell	KIR2DL1	–0.073	0.097	–0.102	0.026	–0.073	0.373	–0.049	0.568
	KIR2DL3	–0.061	0.168	–0.048	0.292	0.04	0.626	0.017	0.845
	KIR2DL4	–0.15	**	–0.153	**	–0.206	0.011	–0.254	*
	KIR3DL1	–0.026	0.551	–0.017	0.716	–0.06	0.462	–0.066	0.443
	KIR3DL2	–0.126	*	–0.149	*	–0.139	0.087	–0.13	0.129
	KIR3DL3	0.034	0.446	0.021	0.654	0.089	0.272	0.056	0.515
	KIR2DS4	–0.086	0.052	–0.092	0.044	–0.059	0.469	–0.063	0.467
Dendritic cell	HLA-DPB1	–0.322	***	–0.326	***	–0.207	0.010	–0.234	*
	HLA-DQB1	–0.252	***	–0.255	***	–0.164	0.043	–0.171	0.045
	HLA-DRA	–0.335	***	–0.348	***	–0.242	*	–0.285	**
	HLA-DPA1	–0.338	***	–0.342	***	–0.22	0.006	–0.23	*
	BDCA-1(CD1C)	–0.235	***	–0.23	***	–0.068	0.404	–0.095	0.268
	BDCA-4(NRP1)	–0.082	0.062	–0.088	0.055	–0.01	0.9	–0.01	0.909
	CD11c (ITGAX)	–0.05	0.26	–0.038	0.402	0.296	**	0.318	**
Th1	T-bet (TBX21)	–0.005	0.901	–0.001	0.988	0.164	0.043	0.136	0.114
	STAT4	0.249	***	0.273	***	0.148	0.067	0.174	0.042
	STAT1	–0.162	**	–0.174	***	–0.037	0.651	–0.053	0.540
	IFN-g (IFNG)	–0.101	0.022	–0.1	0.029	0.061	0.451	0.04	0.640
	TNF-a (TNF)	–0.103	0.020	–0.116	0.011	0.067	0.409	0.049	0.573
Th2	GATA3	–0.174	***	–0.149	*	–0.086	0.293	–0.133	0.120
	STAT6	0.001	0.989	0.005	0.917	0.146	0.072	0.134	0.117
	STAT5A	–0.2	***	–0.22	***	–0.069	0.397	–0.078	0.368
	IL13	0.413	***	0.414	***	0.212	*	0.23	*
Tfh	BCL6	0.095	0.030	0.081	0.075	0.204	*	0.196	0.022
	IL21	0.005	0.917	0.01	0.822	–0.006	0.942	–0.018	0.834
Th17	STAT3	–0.258	***	–0.281	***	0.032	0.691	0.014	0.868
	IL17A	–0.014	0.743	–0.032	0.488	0.056	0.495	0.055	0.524
Treg	FOXP3	0.148	**	0.168	**	–0.043	0.596	–0.049	0.566
	CCR8	–0.034	0.441	–0.024	0.602	–0.119	0.141	–0.133	0.122
	STAT5B	0.044	0.316	0.025	0.589	0.162	0.045	0.151	0.078
	TGFb (TGFB1)	–0.177	***	–0.191	***	0.062	0.448	0.026	0.762
T cell exhaustion	PD-1 (PDCD1)	–0.124	*	–0.115	0.012	0.021	0.798	0.018	0.836
	CTLA4	0.038	0.456	0.058	0.203	–0.045	0.585	–0.059	0.492
	LAG3	0.119	*	0.125	*	0.115	0.158	0.11	0.200
	TIM-3 (HAVCR2)	–0.3	***	–0.325	***	–0.107	0.186	–0.124	0.148
	GZMB	–0.148	0.275	–0.039	0.394	–0.109	0.18	–0.129	0.133

*P < 0.01; **P < 0.001; ***P < 0.0001.

**TABLE 6:** Correlation analysis between MEG3 and relate genes and markers of dendritic cell, monocyte, TAM, and macrophages in GEPIA.

Description	Gene markers	LGG	
		*R*	*P*
Dendritic cell	HLA-DPB1	–0.11	*
	HLA-DQB1	–0.058	0.19
	HLA-DRA	–0.094	*
	HLA-DPA1	–0.08	0.07
	BDCA-1(CD1C)	–0.077	0.081
	BDCA-4(NRP1)	0.16	***
	CD11c (ITGAX)	–0.032	0.47
Monocyte	CD86	–0.19	***
	CD115 (CSF1R)	–0.17	***
TAM	CCL2	0.055	0.21
	CD68	–0.19	***
	IL10	–0.058	0.18
M1 Macrophage	IRF5	–0.13	***
	COX2(PTGS2)	0.036	0.41
M2 Macrophage	VSIG4	–0.13	***
	MS4A4A	–0.11	***

*P < 0.01; ***P < 0.0001.

## Discussion

In the present study, we analyzed the expression level of the lncRNA *MEG3* in glioma and other tumors using the Oncomine database. The results showed that *MEG3* was expressed at a lower level in most cancer types, including gliomas, than in normal tissues. This observation is consistent with the finding in the previous study reporting that *MEG3* might have a tumor suppressive role in gliomas (Ghafouri-Fard and Taheri, 2019). We also found that heterozygous deletion of *MEG3* occurred more frequent than heterozygous amplification in gliomas; mRNA expression of *MEG3* was significantly positively correlated with its CNV in both GBM and LGG groups. Survival analysis showed that the CNV level of *MEG3* had significant correlation with OS and PFS in LGG compared with wild type. Analysis pertaining to the prognostic impact showed that increased expression of *MEG3* was correlated with improved survival in multiple types of human cancers. Through immune infiltration analysis, we found that *MEG3* expression was positively correlated with the degree of CD8^+^ T cell and dendritic cell infiltration in GBM as well as with the degree of dendritic cell, B cell, CD8^+^ T cell, macrophage, CD4^+^ T cell, and neutrophil infiltration in LGG. We further found that infiltration of all these immune cells was significantly associated with LGG prognosis, while only dendritic cell infiltration was significantly associated with GBM prognosis. Furthermore, our analyses showed that levels of immune cell infiltration and diverse immune marker sets were correlated with the level of *MEG3* expression in gliomas, especially in LGG.

The key finding of this study is that *MEG3* expression is correlated with diverse immune cell infiltration levels in gliomas, particularly in LGG. Due to the restrictions imposed by the blood–brain barrier (BBB), the brain has long been considered as an immune privileged organ; nonetheless, the brain is now proposed to be an immunologically distinct organ (Rivest, 2003). Our understanding of the brain tumor microenvironment remains limited. Immune cells constitute an important component of the glioma microenvironments and they can reach up to 50% of the total cell mass in some tumors (Gargini et al., 2020). Gliomas are enriched in diverse immune cell types including neutrophils and myeloid-derived suppressor cells (Thorsson et al., 2018). On the other hand, brain metastases are enriched in lymphocytes and neutrophils. Moreover, brain metastases of different origin showed distinct immune landscape (Klemm et al., 2020). The different immunotherapeutic effects in certain tumors may be attributed to differences in the disease- and cell-type-specific tumor microenvironments. A previous study showed that downregulation of *MEG3* promotes angiogenesis after ischemic brain injury (Liu et al., 2017). BBB disruption following stroke promotes inflammation by enabling leukocytes, T cells, and other immune cells to migrate across the BBB (Huang et al., 2020), which may indirectly explain the potential mechanism of the role of *MEG3* in the immune microenvironment. The tumor immune microenvironment influences cancer immune escape, response to immunotherapy, and the survival rate of patients (Zhang et al., 2020). The observed correlation between *MEG3* and the expression of certain immunological marker genes suggests that, in gliomas, *MEG3* may interact with infiltrated immune cells within the tumor microenvironment. Of note, evidence that any therapeutic intervention administered to glioma patients has a major effect on survival is lacking. Our analysis would help in an efficient implementation of immunotherapy as a part of the standard care for patients with glioma.

Functional studies indicate that lncRNAs may act as oncogenes or tumor suppressors in various cancers (Shi et al., 2013). The results obtained from PrognoScan database analysis were consistent with the proposed tumor suppressor role for *MEG3*. Several studies have shown a correlation between *MEG3* expression and patients’ survival. Low *MEG3* expression level was correlated with poor prognosis (Zhao et al., 2018). A recent meta-analysis has verified that low expression of *MEG3* is significantly associated with poor prognosis for patients with different types of cancer (Binabaj et al., 2018), although GEPIA database analysis showed that changes in *MEG3* expression had no significant effect on the OS and DFS in both LGG and GBM. Our study showed that immune cell infiltration has close correlation with prognosis in glioma patients. The association between *MEG3* expression and immune cell infiltration indirectly indicated that *MEG3* may be a useful predictor of glioma patient survival. Furthermore, using the PrognoScan database, we observed that *MEG3* expression was significantly correlated with the prognosis of a total of nine cancer types, including gliomas. These findings warrant validation in a larger cohort as previous sample size was limited. *MEG3* inhibits cancer progression through several independent mechanisms, including regulation of the tumor suppressor genes p53 and Rb (Li et al., 2016; Lyu et al., 2017), inhibition of angiogenesis (Gordon et al., 2010), acting as a competitive endogenous (ce)RNA (Qin et al., 2017; Zhang et al., 2017; Zhang and Guo, 2019; Gong and Huang, 2020), and induction of EMT and invasion *via* autophagy (Gong and Huang, 2017; Yang et al., 2020). Although the role and prognostic value of the dysregulation of *MEG3* in gliomas has not yet been fully evaluated, the current findings suggest that *MEG3* may regulate tumor progression *via* immune-based mechanisms. Further studies are needed to establish its exact molecular mechanism in the progression of gliomas.

The tumor database-based studies have advantages of a larger sample size and higher reliability over traditional studies; such studies also provide a preliminary foundation for further studies. Although our study provides insights into understanding the potential role of *MEG3* in tumor immunology and its use as a cancer biomarker, it is limited in terms of lack of *in vitro* and *in vivo* experiments to validate the relationship between *MEG3* expression and infiltration of immune cells in gliomas. Therefore, further research is needed to verify the role of *MEG3* in gliomas.

## Conclusion

Taken together, the current findings suggest that the expression of *MEG3* might serve as a possible prognostic biomarker and potential immunotherapeutic target for gliomas.

## Data Availability Statement

The datasets presented in this study can be found in online repositories. The names of the repository/repositories and accession number(s) can be found in the article/Supplementary Material.

## Author Contributions

XX analyzed the data and wrote the manuscript. YY reviewed the data and manuscript. ZZ and YS analyzed the data, edited the manuscript, and supervised the study. All authors contributed to the article and approved the final submitted version of the manuscript.

## Conflict of Interest

The authors declare that the research was conducted in the absence of any commercial or financial relationships that could be construed as a potential conflict of interest.

## Footnotes

1 https://www.oncomine.org/resource/login.html

2 https://cistrome.shinyapps.io/timer/

3 http://bioinfo.life.hust.edu.cn/web/GSCALite/

4 www.prognoscan.org/

5 http://gepia.cancer-pku.cn/index.html

## Supplementary Material

Supplementary Figure 1 MEG3 expression levels in GBM and LGG in the GEPIA database.

Supplementary Figure 2 Kaplan-Meier survival curves comparing the low and high expression of *MEG3* in LGG and GBM using the GEPIA database.
