# Supplementary figures and images for: Prognostic Value of MEG3 and Its Correlation With Immune Infiltrates in Gliomas
Source: Front Genet. 2021 Jun 16;12:679097. doi: 10.3389/fgene.2021.679097 (PMC8242350; doi:10.3389/fgene.2021.679097)

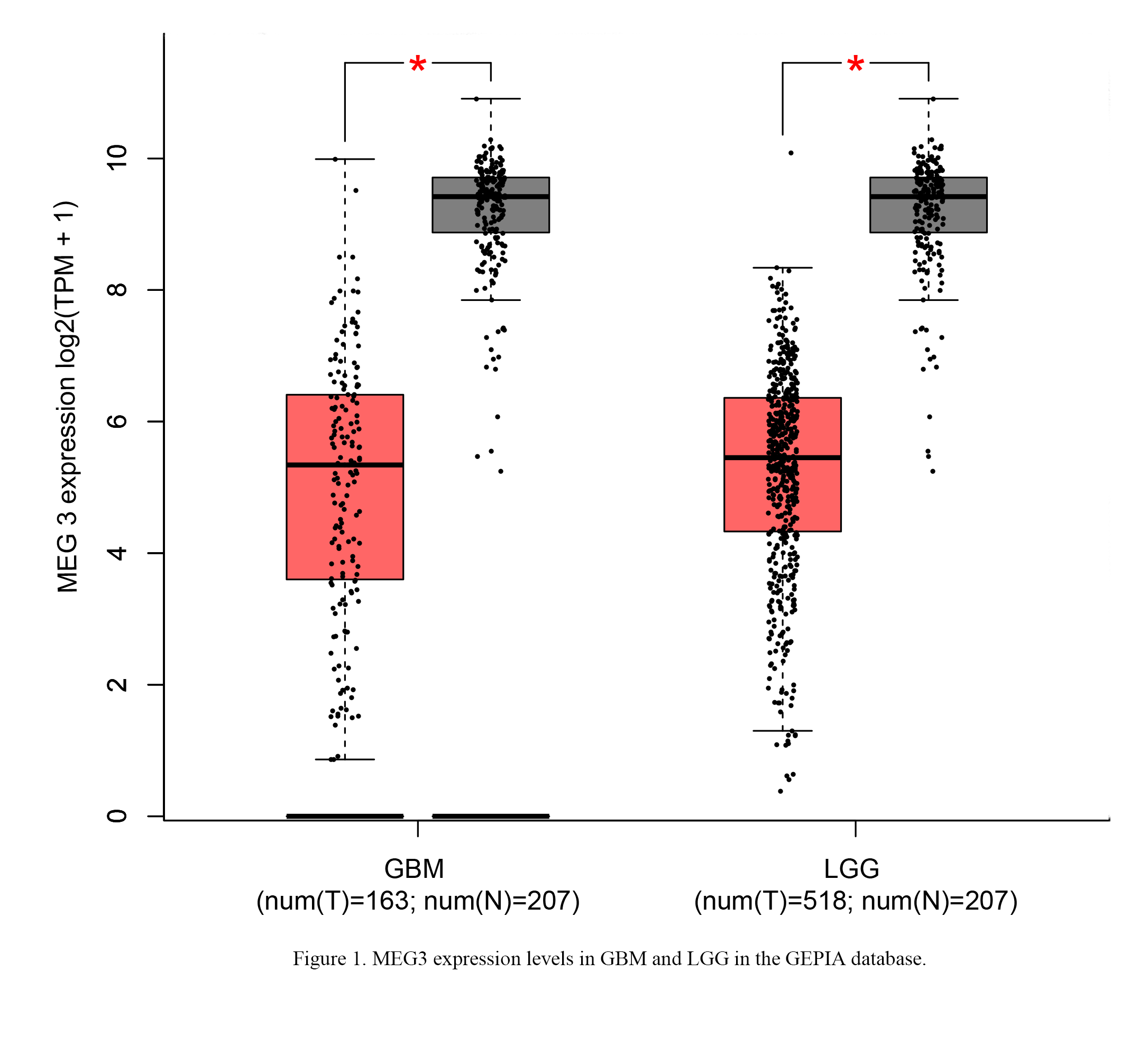

Supplement: Supplementary Figure 1 — MEG3 expression levels in GBM and LGG in the GEPIA database. [file Image_1.TIF]

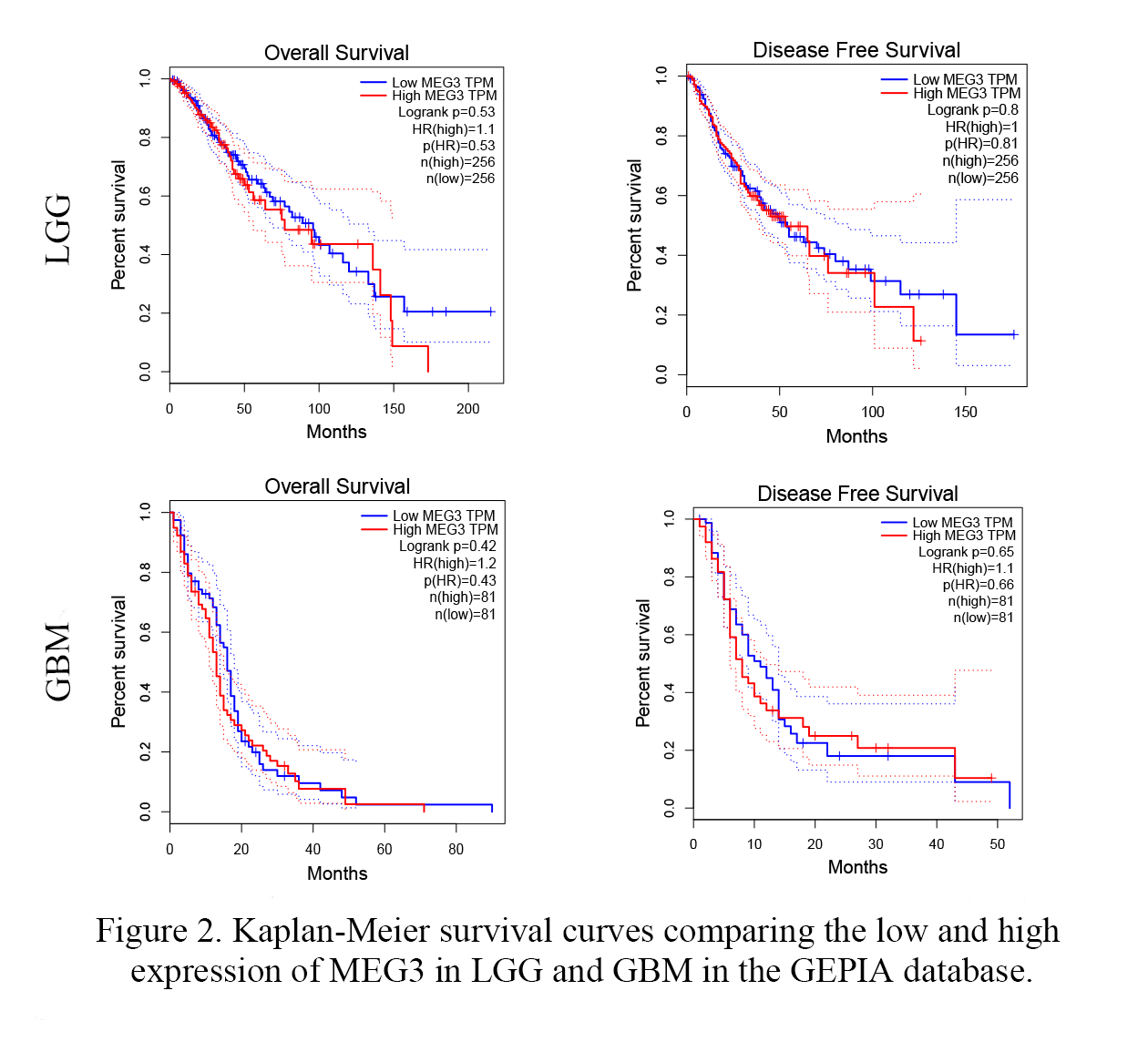

Supplement: Supplementary Figure 2 — Kaplan-Meier survival curves comparing the low and high expression of MEG3 in LGG and GBM using the GEPIA database. [file Image_2.TIF]
